# O-Arm- and Guide-Device-Assisted Personalized Percutaneous Kyphoplasty for Thoracolumbar Osteoporotic Vertebral Compression Fractures

**DOI:** 10.3390/jpm13040595

**Published:** 2023-03-29

**Authors:** Hongwei Wang, Bin Zheng, Hongwen Gu, Yuanhang Zhao, Da Liu, Hailong Yu, Liangbi Xiang

**Affiliations:** 1 Department of Orthopedics, General Hospital of Northern Theater Command of Chinese PLA, Shenyang 110016, China; cplawhw@163.com (H.W.);; 2 Department of Orthopedics, General Hospital of Western Theater Command of Chinese PLA, Chengdu 610083, China

**Keywords:** O-arm, guide device, percutaneous kyphoplasty, osteoporotic vertebral compression fracture, planning

## Abstract

With the ageing of the global population, the incidence of osteoporotic vertebral compression fractures (OVCFs) is increasing. To assess the safety and efficacy of O-arm- and guide-device-assisted personalized percutaneous kyphoplasty (PKP) for treating thoracolumbar OVCFs, a total of 38 consecutive thoracolumbar OVCF patients who underwent bilateral PKP assisted with an O-arm and a guide device (O-GD group, *n* = 16) or traditional fluoroscopy (TF group, *n* = 22) from January 2020 to December 2021 were retrospectively reviewed, and their epidemiologic, clinical and radiological outcomes were analysed. The operation time was significantly decreased (*p* < 0.001) in the O-GD group (38.3 ± 12.2 min) compared with the TF group (57.2 ± 9.7 min). The number of intraoperative fluoroscopy exposures was significantly decreased (*p* < 0.001) in the O-GD group (31.9 ± 4.5) compared with the TF group (46.7 ± 7.2). Intraoperative blood loss was significantly decreased (*p* = 0.031) in the O-GD group (6.9 ± 2.5 mL) compared with the TF group (9.1 ± 3.3 mL). No significant difference (*p* = 0.854) in the volume of injected cement was observed between the O-GD group (6.8 ± 1.3 mL) and the TF group (6.7 ± 1.7 mL). Both the clinical and radiological outcomes, including the visual analogue scale score for pain, Oswestry Disability Index and anterior height and local kyphotic angle of the fractured vertebrae, were significantly improved at the postoperative and final follow-up but did not differ between the two groups. The incidence of cement leakage and refracture of the vertebral body was similar in the two groups (*p* = 0.272; *p* = 0.871). Our preliminary study demonstrated that O-GD-assisted PKP is a safe and effective procedure that presents a significantly shorter operation time, fewer intraoperative fluoroscopy exposures and less intraoperative blood loss than the TF technique.

## 1. Introduction

With the ageing of the global population, the incidence of osteoporotic vertebral compression fractures (OVCFs) is increasing. OVCFs significantly decrease quality of life due to pain, functional disorders and spinal deformities [1]. Percutaneous kyphoplasty (PKP) is a minimally invasive surgery that has been found to be a safe and effective therapy for OVCFs. Currently, the standard technique for PKP is a bilateral approach [2,3].

Many advanced techniques have been used to assist in PKP [4,5,6,7,8,9,10,11,12,13], including surgical robots [4,5,6,7,8], real-time ultrasound volume navigation [9], DynaCT [10] and O-arm navigation [11,12,13]. However, these technologies are expensive and/or have high technical requirements; thus, they cannot be widely used in primary hospitals. We have given much attention to the preoperative planning [14,15] and 3D printing technologies [16,17,18,19,20] used in percutaneous vertebroplasty (PVP)/PKP, and previous studies have shown that PVP with preoperative puncture design is not only reasonably safe and effective for OVCFs but also involves less radiation, shorter operation times and fewer complications than traditional PVP [14,15]. Our research has indicated that unilateral puncture using a novel 3D-printed guide device is a safe and effective technique for OVCFs and is associated with fewer intraoperative fluoroscopy exposures and shorter operation times [21]. During PKP, the conventional methods used for Jamshidi needle placement and guidewire advancement into the pedicle require numerous radiographic images. With the help of our novel intradermal locator, the best bone puncture point could be accurately located. After determining the bone puncture point, the angle of the puncture trajectory could be adjusted using the angle-measuring device, and repeat fluoroscopy to determine the relationship between the needle and the pedicle was avoided [21].

In recent years, O-arm navigation, which provides very helpful intraoperative imaging, has been widely used in most spinal surgeries [11,12,13,22,23,24,25,26,27]. O-arm navigation can provide an accurate needle entry point and puncture trajectory, as well as an accurate location of vertebral lesions. However, the O-arm navigation technique requires general anaesthesia, and there is an increased risk during and after general anaesthesia in elderly patients. At the same time, the need to install a reference frame that is fixed to the adjacent spinous process or superoposterior iliac crest during the operation may increase the patient’s pain and aggravate postoperative pain [11,12].

In the proposed method, the accuracy of the personalized skin puncture point and trajectory are confirmed by preoperative planning using the O-arm, the bone puncture point and insertion angle of the trajectory are determined during the operation using our novel guide device, which is manufactured through 3D printing technology, and then the operation is completed with the aid of fluoroscopy. The main purpose of this retrospective study was to evaluate the safety and efficacy of O-arm- and guide-device-assisted PKP for treating thoracolumbar OVCFs compared with the traditional PKP technique without the assistance of the O-arm and guide device.

## 2. Materials and Methods

### 2.1. Patients

From January 2020 to December 2021, 38 elderly patients (≥60 years old) diagnosed with single-level OVCFs underwent bilateral PKP. The patients were divided into two groups: bilateral PKP assisted with an O-arm and guide device (O-GD group, *n* = 16) and traditional fluoroscopy (TF group, *n* = 22). Preoperative demographic, clinical and radiologic characteristics, operative details and clinical and radiologic outcomes at 1 day and 12 months postoperatively were collected.

The inclusion criteria were as follows: (1) a single-level fracture in the thoracolumbar vertebrae (T11-L2) identified through preoperative radiological findings and tapping pain at the spinal process of the fractured vertebral body; (2) a preoperative visual analogue scale (VAS) pain score over 5; (3) a new fracture without any previous fractures of the vertebrae, as determined through preoperative radiological findings; (4) a T value ≤ −2.5, as determined by a bone mineral density (BMD) examination of the lumbar vertebrae; and (5) an intact posterior vertebral wall. The exclusion criteria were as follows: (1) a history of previous vertebral augmentation; (2) a pathological fracture caused by a tumour or spine infection; and (3) comorbidities involving thyroid dysfunction. Patients were treated by the same senior surgeon (H.W.), who performed the surgical techniques at the same institution during the study period. The senior surgeon had performed more than 200 kyphoplasty procedures before the study. Preoperative, 1-day postoperative and 12-month postoperative VAS scores and Oswestry Disability Index (ODI) values were determined by an orthopaedic resident. X-ray, CT or MRI examinations were performed preoperatively, 1 day postoperatively and 12 months postoperatively by a radiologist. The orthopaedic resident and radiologist were blinded to the grouping of the patients. The study protocol was approved by the ethics committee of the General Hospital of the Northern Theater Command (Y-2021-025).

### 2.2. Guide Device

**Figure 1:**
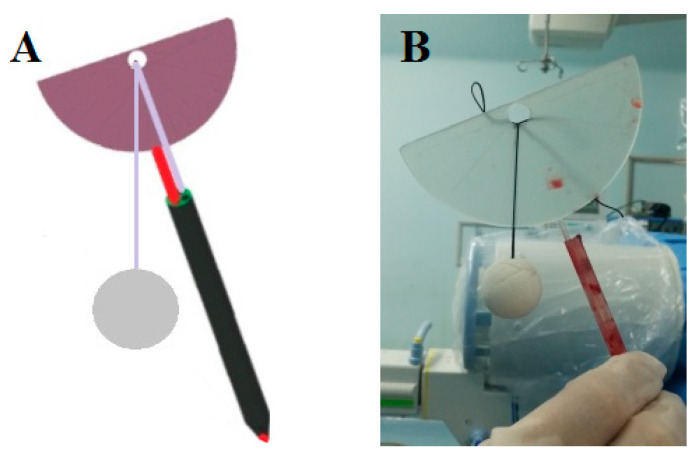
Schematic diagram and intraoperative image showing the guide device. (**A**) Schematic diagram of the guide device. (**B**) Intraoperative image showing the 3D-printed guide device.

The novel guide device (Figure 1) includes a puncture needle or Kirschner wire (red line in Figure 1A), an intradermal locator (black tube in Figure 1A) and an angle-measuring device (pink semicircle in Figure 1A) [21]. The novel guide device was created by reverse engineering and rapid prototyping techniques (Figure 1B).

### 2.3. Surgical Procedures

**Figure 2:**
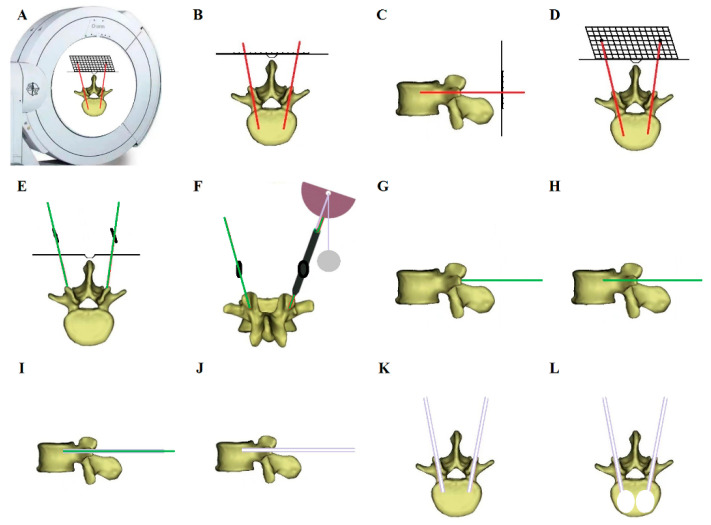
Schematic diagram showing the surgical procedure. (**A**) Three-dimensional CT scans of the fractured vertebral body and body surface locator were carried out using the O-arm. (**B**–**D**) CT scans were used to reconstruct a 3D image and perform preoperative planning. (**E**) The entry point on the skin was determined using the body surface locator before the operation. (**F**) The intradermal locator was placed along the Kirschner wire, and then the guide device was deflected according to the planned trajectory, which could be observed using the angle-measuring device. (**G**–**K**) A dilating cannula and a working cannula were inserted to establish the working channel. (**L**) Cement was injected into the vertebra.

**Figure 3:**
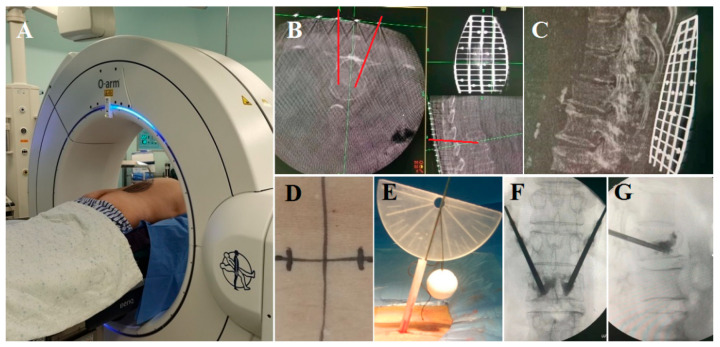
Intraoperative images of a 68-year-old woman with an L1 vertebral compression fracture. (**A**–**C**) Three-dimensional CT scans carried out using the O-arm were used to perform preoperative planning. (**D**) The entry point on the skin was determined using the body surface locator before the operation. (**E**) A 3D-printed guide device was used to determine the bone puncture point and insertion angle of the trajectory during the operation. (**F**,**G**) Cement was injected into the vertebra through bilateral PKP.

**Figure 4:**
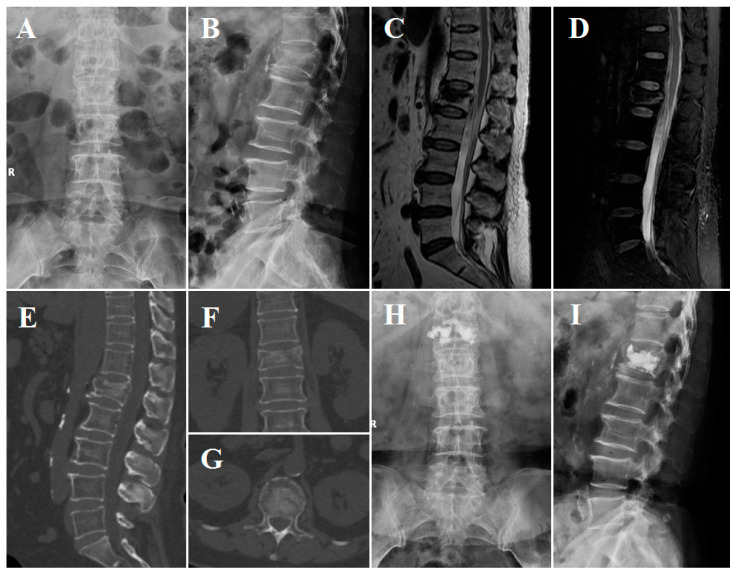
Imaging obtained from the patient described above. (**A**–**D**) Preoperative X-rays and MRI. (**E**–**G**) Preoperative CT showing fissures in the lateral and upper endplates of the fractured vertebral body. (**H**,**I**) Postoperative lumbar X-rays obtained one day after PKP.

All patients were placed in the prone position on an operating table. In the TF group, the surgical segment was under fluoroscopic guidance. Then, the needle entry point was confirmed by repeat fluoroscopy. After puncture via the pedicle was finished, the working canal was inserted, and cement was injected. Next, continuous fluoroscopy was used to verify the position of the cement and to stop injection when the vertebra had been filled with an appropriate amount of cement. In the O-GD group, the body surface locator was placed on the skin above the fractured vertebral body to ensure that the skin puncture point was within the range of the locator, and three-dimensional CT scans of the fractured vertebral body and body surface locator were carried out using the O-arm (Figure 2A). With the help of the O-arm device, axial, coronal and sagittal CT scans were obtained preoperatively to perform 3D reconstruction, locate the collapsed vertebral body and perform preoperative planning (Figure 2B–D). Therefore, the entry point on the skin could be determined by the body surface locator before the operation (Figure 2E). After disinfection, dressing and local anaesthesia, a Kirschner wire was percutaneously inserted through the entry point on the skin to dock at the approximate position of the lateral margin of the facet joint, and the intradermal locator was placed along the Kirschner wire. Then, the guide device was deflected along the transverse plane, and the Kirschner wire was inserted into the body according to the planned trajectory, which could be observed using the angle-measuring device intraoperatively (Figure 2F). Subsequently, a dilating cannula and a working cannula were inserted to establish the working channel (Figure 2G–K). The following procedural processes were the same as those in traditional PKP surgery, and finally, the cement was injected into the vertebra (Figure 2L). Cases illustrating the surgical procedures and perioperative imaging data are shown in Figure 3 and Figure 4.

### 2.4. Outcome Measures

The following data were collected by reviewing medical records: patient age, sex, body mass index, bone mass density (T score), fracture level and perioperative parameters, including the operation time, intraoperative bleeding, number of intraoperative fluoroscopy exposures, volume of injected cement, length of hospital stay and hospitalization cost. The operation time was recorded from the time the patient was placed in the prone position until the cement push rod was removed after cement injection. The VAS score for back pain and the ODI were routinely determined for all patients. Clinical outcomes were compared between the groups. Meanwhile, preoperative, 1-day postoperative, 1-month postoperative and 12-month postoperative VAS scores and ODI values were compared. Radiological outcomes were compared through X-rays or CT scans taken preoperatively, 1 day postoperatively and 12 months postoperatively, which were reviewed for determination of the anterior height of the fractured vertebra (AVH), the local kyphotic angle (LKA) of the fractured vertebra and the extent of reduction and correction. The safety of PKP was assessed by evaluating surgical complications, including cement leakage, nerve injury and postoperative infection. Postoperative infection was assessed based on serum leukocytes and inflammatory markers, including C-reactive protein and the erythrocyte sedimentation rate.

### 2.5. Statistical Analysis

Statistical analyses were carried out using SPSS 24.0 (IBM Corporation, Armonk, NY, USA). Nominal variables are expressed as numbers (percentages). The chi-square test or Fisher’s exact test was used to compare frequency data, the Shapiro–Wilk method was used to test the normality of measurement data, ANOVA was used for the comparison of measurement data with a normal distribution, and the LSD method was used for post hoc analysis. The Kruskal–Wallis H test with the Bonferroni method was used for the comparison of measurement data with a partial distribution between groups. Other characteristics were compared between the two groups via an independent-sample *t* test. The AVH, LKA, VAS score and ODI within groups were compared via a paired *t* test. The significance level was set to 0.05.

## 3. Results

### 3.1. General Information

The mean age of all patients was 70.6 ± 8.0 years (60–89 years). No significant differences were observed in age, sex, BMI, BMD, fractured vertebrae or mean follow-up duration between the two groups (Table 1).

### 3.2. Perioperative Outcome Comparison

The operation time was significantly decreased (*p* < 0.001) in the O-GD group (38.3 ± 12.2 min) compared with the TF group (57.2 ± 9.7 min). The number of intraoperative fluoroscopy exposures was significantly decreased (*p* < 0.001) in the O-GD group (28.9 ± 4.5) compared with the TF group (46.7 ± 7.2). Intraoperative blood loss was significantly decreased (*p* = 0.031) in the O-GD group (6.9 ± 2.5 mL) compared with the TF group (9.1 ± 3.3 mL). No significant difference (*p* = 0.854) was observed in the volume of injected cement between the O-GD group (6.8 ± 1.3 mL) and the TF group (6.7 ± 1.7 mL) (Table 2).

### 3.3. Clinical and Radiological Outcome Comparison

Both clinical and radiological outcomes, such as the VAS score, ODI and AVH and LKA of the fractured vertebra, were improved significantly at the postoperative and final follow-up but did not differ between the two groups (Table 3).

### 3.4. Complication Comparison

Regarding complications, the incidence of cement leakage was similar in the two groups (*p* = 0.272; *p* = 0.871). CT scans showed asymptomatic cement leakage in 12 patients after PKP surgery, with 9 in the TF group (40.9%) and 3 in the O-GD group (18.8%). During the follow-up period, no cases of neurological deficit, infection or pulmonary embolism were observed (Table 4).

## 4. Discussion

In recent years, O-arm navigation, which provides very helpful intraoperative imaging, has been widely used in most spinal surgeries [11,12,13,22,23,24,25,26,27]. O-arm navigation can provide an accurate needle entry point and puncture trajectory, as well as an accurate location of vertebral lesions. Fujiwara et al. have shown that O-arm navigation enables a safe and accurate osteotomy to be performed in musculoskeletal tumour resection [22]. Compared with fluoroscopy, O-arm navigation allows endoscopic transforaminal lumbar interbody fusion (Endo-TLIF) surgery to be performed more quickly with less radiation exposure and similar clinical outcomes [23]. O-arm navigation also has a good effect on improving the accuracy of screw insertion and clinical outcomes in adult deformity, occipitocervical fusion and multiple lumbar fusion surgery [24,25,26,27].

O-arm navigation techniques also play an important role in PKP [11,12]. O-arm navigation-assisted unilateral PKP is a safe and effective minimally invasive method for the treatment of Kümmell’s disease. This surgery can effectively relieve pain, restore physiological spinal curvature and improve functional status [11]. Another study revealed that PKP assisted with O-arm navigation is a safe and effective procedure that can be applied for midthoracic OVCFs and significantly restore radiological and clinical outcomes, with a low rate of complications [12]. However, the O-arm navigation technique requires general anaesthesia, which can increase the risk during and after general anaesthesia in elderly patients. At the same time, the need to install a reference frame that is fixed to the adjacent spinous process or superoposterior iliac crest during the operation may increase the patient’s pain and aggravate postoperative pain [11,12].

In previous studies, preoperatively designed PVP involved less radiation, a shorter operation and fewer complications than traditional PVP [14,15]. Our previous research has also indicated that unilateral PKP using a novel 3D-printed guide device is a safe and effective technique for OVCFs [21]. The 3D-printed guide devices, including an intradermal locator and an angle-measuring device, were used to accurately determine the entry point and angle of trajectory. During PKP, the conventional methods used for Jamshidi needle placement and guidewire advancement into the pedicle require numerous radiographic images. With the help of our novel intradermal locator, the best bone puncture point could be accurately located. After determining the bone puncture point, the angle of the puncture trajectory could be adjusted using the angle-measuring device, and repeat fluoroscopy to determine the relationship between the needle and the pedicle was avoided [21]. However, there may be errors when the patient undergoes supine CT imaging in the CT examination room and enters the operating room in the prone posture for surgery.

In the current study, we performed preoperative planning using the O-arm in the operating room with the patient in the prone position and performed the operation using our 3D-printed guide device with the patient in the same place and the same posture, thereby avoiding errors caused by changes in the patient’s posture. This approach allows for the individualized design of the skin entry point and puncture path before surgery in the operating room and guidance of the puncture path using the 3D-printed guide device during the puncture process. Our results indicate that O-GD-assisted PKP is a safe and effective procedure that presents significantly shorter operation times, fewer intraoperative fluoroscopy exposures and less intraoperative blood loss than the TF technique. However, it is important to note that fluoroscopy and 3D reconstruction using the O-arm are not included in the calculation. We only needed to use the O-arm once during the operation, and in the future, we could use O-arm fluoroscopy and 3D reconstruction to replace preoperative 3D CT, which could significantly reduce the patient’s radiation exposure throughout the treatment process.

The most common complication after vertebroplasty or kyphoplasty is cement leakage (14–18%) [28,29,30]. In the current study, bone cement leakage occurred in 18.8% of the patients in the O-GD group and 40.9% of the patients in the TF group, but there was no significant difference between the two groups. Bone cement usually infiltrates the epidural space, and intervertebral disc, anterior and lateral leakage and can affect the postoperative prognosis of the patient. In the TF group, anterior leakage occurred in one patient, lateral leakage occurred in two patients, and intervertebral leakage occurred in six patients. In the O-GD group, lateral leakage occurred in three patients. The higher frequency of cement leakage (40.9%), especially intervertebral leakage, in the TF group was attributed to the bone cement injection site being too close to the fractured endplate. To avoid bone cement leakage, preoperative CT scans should be performed, and whether the surrounding wall and endplate of the vertebral body are damaged should be confirmed. Attention should be given to the puncture angle and target site, and a small piece of gelatine sponge could be placed at the site where the bone cement is to be injected if there is a crack in the lateral wall of the vertebral body near the injection site. The bone cement injection should be slowed or stopped if the cement diffuses to the posterior margin of the vertebral body. The frequency of cement leakage was decreased in the O-GD group because we confirmed the accuracy of the skin puncture point and trajectory, which were sufficiently far from the fractured endplate, through preoperative planning using the O-arm. During the follow-up period, no cases of neurological deficit, infection or pulmonary embolism were observed. These results indicate that it is safe to perform PKP surgery with the assistance of an O-arm and 3D-printed guide device.

In the current study, we set strict inclusion and exclusion criteria. Osteoporosis was an important criterion in our study design, and it was important to specify the BMD so we could choose PKP to treat patients while ensuring no differences in basic characteristics. The effect of BMD in this study was excluded as much as possible. We do not deny that fractures caused by thyroid dysfunction can be treated with PKP. The pathogenesis of an osteoporotic fracture caused by thyroid dysfunction is different from that of a simple osteoporotic fracture. We excluded patients with osteoporotic fractures due to thyroid dysfunction and patients with a VAS score less than or equal to 5 to minimize differences in basic characteristics and allow subsequent clinical and radiological follow-up evaluations to be performed.

This study also has some limitations. First, this was a retrospective study and may have been affected by selection bias, which may be accompanied by a lower level of evidence than in randomized controlled trials. Additionally, the novel guide device was only used to assist surgery and was not permanently implanted in the body. After obtaining ethics approval from the ethics committee of the General Hospital of the Northern Theater Command, we used O-arm assistance and the novel guide-device-assisted technique to treat thoracolumbar OVCFs. However, the degree of bias may be limited because we used strict inclusion and exclusion criteria. Second, the sample size was relatively small, the follow-up time was relatively short, and long-term patient complications were not evaluated, which may affect the accuracy of the conclusions. Third, it is important to compare the radiation dose between the two groups, and we evaluated the number of fluoroscopy exposures to indirectly compare the radiation dose, which may not yield an accurate estimate; however, this measure has been used in prior studies and thus has some comparative value [21,31,32]. Future prospective randomized controlled studies involving a larger sample of patients with longer follow-up times and more radiation dose data could address these limitations.

## 5. Conclusions

With the help of an O-arm and 3D-printed guide device, the planned skin and bone puncture points and the puncture trajectory were easily determined, and repeat fluoroscopy to determine the relationship between the needle and the pedicle was avoided. The O-GD group presented with significantly shorter operation times, fewer intraoperative fluoroscopy exposures and less intraoperative blood loss, and the VAS score, ODI and AVH and LKA of the fractured vertebra were improved significantly. During the follow-up period, no cases of neurological deficit, infection or pulmonary embolism were observed.

## Figures and Tables

**Table 1:** Comparison of characteristic parameters between the 2 groups.

Characteristic		TF Group (*n* = 22)	O-GD Group (*n* = 16)	*p*
Male/female		7/15	4/12	0.924
Mean age (years)		69.9 ± 7.8	71.6 ± 8.3	0.520
BMI (kg/m^2^)		22.3 ± 2.7	23.9 ± 2.1	0.054
BMD		−3.0 ± 0.5	−3.2 ± 0.6	0.377
Mean follow-up duration (months)		15.5 ± 3.2	15.6 ± 5.1	0.991
Fractured vertebra				
	T11	3	3	0.623
	T12	6	7	
	L1	9	4	
	L2	4	2	

**Table 2:** Comparison of changes in perioperative parameters between the 2 groups.

Characteristic	TF Group (*n* = 22)	O-GD Group (*n* = 16)	*p*
Operation time (mins)	57.2 ± 9.7	38.3 ± 12.2	<0.001
Intraoperative blood loss (mL)	9.1 ± 3.3	6.9 ± 2.5	0.031
Intraoperative fluoroscopy exposures	46.7 ± 7.2	28.9 ± 4.5	<0.001
Volume of injected cement (mL)	6.8 ± 1.3	6.7 ± 1.7	0.854
Postoperative hospital stay (days)	1.5 ± 1.4	1.1 ± 0.5	0.326
Hospitalization cost (×10^4^ CNY)	3.4 ± 0.2	3.5 ± 0.2	0.107

**Table 3:** Comparison of changes in clinical and radiographic parameters between the 2 groups.

Characteristic		TF Group (*n* = 22)	O-GD Group (*n* = 16)	*p*
VAS score				
	Preoperative	7.2 ± 0.9	7.4 ± 0.6	0.416
	1 day postoperative	3.5 ± 0.8	3.1 ± 0.8	0.139
	1 month postoperative	2.9 ± 0.9	2.3 ± 0.9	0.052
	12 months postoperative	1.7 ± 0.6	1.4 ± 0.5	0.108
ODI				
	Preoperative	76.1 ± 10.4	78.7 ± 3.9	0.303
	1 day postoperative	37.5 ± 6.1	39.5 ± 4.5	0.285
	1 month postoperative	28.4 ± 3.7	28.0 ± 3.9	0.772
	12 months postoperative	15.8 ± 4.4	16.4 ± 3.1	0.670
AVH (mm)				
	Preoperative	22.3 ± 5.5	21.0 ± 5.3	0.452
	1 day postoperative	27.8 ± 4.5	26.8 ± 5.1	0.557
	12 months postoperative	26.7 ± 4.4	25.3 ± 4.4	0.341
LKA (°)				
	Preoperative	11.5 ± 4.9	11.5 ± 6.1	0.988
	1 day postoperative	7.6 ± 3.7	7.1 ± 4.9	0.736
	12 months postoperative	8.8 ± 3.9	8.1 ± 5.0	0.652

**Table 4:** Comparison of complications between the 2 groups.

Characteristic	TF Group (*n* = 22)	O-GD Group (*n* = 16)	*p*
Refracture (*n*)	0	1 (6.3%)	0.871
Cement leakage (*n*)	9 (40.9%)	3 (18.8%)	0.272
Neurological deficit (*n*)	0	0	-
Pulmonary embolism (*n*)	0	0	-
Infection (*n*)	0	0	-

## Data Availability

The data that support the findings of this study are available from the corresponding author on special request.
